# Targeted Regression of Hepatocellular Carcinoma by Cancer-Specific RNA Replacement through MicroRNA Regulation

**DOI:** 10.1038/srep12315

**Published:** 2015-07-20

**Authors:** Juhyun Kim, Ranhui Won, Guyee Ban, Mi Ha Ju, Kyung Sook Cho, Sang Young Han, Jin-Sook Jeong, Seong-Wook Lee

**Affiliations:** 1 Department of Molecular Biology, Institute of Nanosensor and Biotechnology, and Research Institute of Advanced Omics, Dankook University, Yongin 448-701, Republic of Korea; 2 Department of Pathology and Immune-network Pioneer Research Center, Dong-A University College of Medicine, Busan 602-714, Republic of Korea; 3 Department of Internal Medicine, Dong-A University College of Medicine, Busan 602-714, Republic of Korea

## Abstract

Hepatocellular carcinoma (HCC) has a high fatality rate and limited therapeutic options with side effects and low efficacy. Here, we proposed a new anti-HCC approach based on cancer-specific post-transcriptional targeting. To this end, *trans*-splicing ribozymes from *Tetrahymena* group I intron were developed, which can specifically induce therapeutic gene activity through HCC-specific replacement of telomerase reverse transcriptase (TERT) RNA. To circumvent side effects due to TERT expression in regenerating liver tissue, liver-specific microRNA-regulated ribozymes were constructed by incorporating complementary binding sites for the hepatocyte-selective microRNA-122a (miR-122a), which is down-regulated in HCC. The ribozyme activity *in vivo* was assessed in mouse models orthotopically implanted with HCC. Systemic administration of adenovirus encoding the developed ribozymes caused efficient anti-cancer effect and the least hepatotoxicity with regulation of ribozyme expression by miR-122a in both xenografted and syngeneic orthotopic murine model of multifocal HCC. Of note, the ribozyme induced local and systemic antitumor immunity, thereby completely suppressing secondary tumor challenge in the syngeneic mouse. The cancer specific *trans*-splicing ribozyme system, which mediates tissue-specific microRNA-regulated RNA replacement, provides a clinically relevant, safe, and efficient strategy for HCC treatment.

Hepatocellular carcinoma (HCC) is the most common primary malignancy of the liver in adults, generally followed by liver cirrhosis, and is caused by risk factors such as the hepatitis B and C virus, HIV, obesity, diabetes, alcohol intake, or environmental exposures. More than half a million individuals are diagnosed with HCC per year, making it the third highest in cancer mortality worldwide^1^. A number of strategies such as surgery, chemotherapy, molecular targeted therapy, radiation, and liver transplantation have been utilized for the treatment of HCC, however, most of the therapeutic treatments have a low response rate and are accompanied with significant side effects^2^,^3^,^4^. Moreover, the high fatality rate of HCC has not yet improved, primarily because of uncontrolled metastasis, liver failure, and its resistance to therapeutic agents. To overcome these clinical shortcomings, genetic therapy has been considered as a new therapeutic approach for HCC.

Gene therapy for cancer treatment has focused on enhancing both efficacy and safety, leading to efficient suppression of cancer cells without harming normal cells^5^. However, the ideal of accurate and effective cancer cell-specific targeting remains an important challenge^6^. The group I intron from the *Tetrahymena* based *trans*-splicing ribozyme has been shown to target and cleave a substrate RNA and *trans*-ligate the 3′exon of the ribozyme onto the downstream U nucleotide of the cleaved target RNA in cells^7^,^8^. This *trans*-splicing ribozyme has been used for therapeutic applications, such as in the treatment of human genetic or malignant diseases^9^,^10^,^11^,^12^. Previously, we validated that the *trans*-splicing ribozyme could be a potent anticancer agent through selective targeting and RNA replacement of the transcripts dominantly expressed in cancer cells. We developed a *trans*-splicing ribozyme targeting human telomerase reverse transcriptase (hTERT) RNA, and evidenced that the ribozyme could efficiently induce suicide gene activity through target-specific *trans*-splicing reaction not only in cells, but also in tumor xenografts models when treated with pro-drugs^13^,^14^,^15^,^16^. However, the proliferative normal liver cells of patients with cirrhosis express low levels of hTERT during the premalignant or later stages of hepatocarcinogenesis^17^. Thus, the TERT-targeted therapy could affect proliferative normal liver tissue as well as the diseased tissue. Consequently, novel therapeutic strategies should focus on specifically targeting cancerous liver cells whilst sparing the normal, healthy liver cells for clinical relevance.

The important roles of small and noncoding microRNAs (miRNAs) have been demonstrated in cancer^18^,^19^. Dysregulation of miRNA expression occurs in cancer, and certain miRNAs can function as oncogenes^20^ or tumor suppressor^21^. Recently, endogenous miRNAs have been exploited in cell-specific targeting by regulating transgene expression according to tissue, lineage, differentiation, and disease state through the insertion of miRNA target sites into the 3′-untranslated region (UTR) of an expression cassette^22^. MiRNA-122a (miR-122a) is a liver-specific miRNA that plays an important role in physiological processes such as lipid metabolism^23^, stress response^24^, and tumor suppression^25^. The level of miR-122a is extremely high in normal hepatocytes, but it is either silent or expressed at low levels in most HCC and transformed cell lines^26^. Therefore, miR-122a could be utilized for selective transgene expression in tissues other than hepatocytes.

In this study, we combined post-transcriptional regulation with an RNA replacement strategy by developing TERT-targeting *trans*-splicing ribozymes regulated by liver-specific miR-122a (Fig. 1) and evaluated the cellular and *in vivo* specificity and efficacy as a tool for targeted HCC gene therapy to address the cancer-selectivity challenge raised by the TERT-targeting approach. Viral vectors encoding the ribozymes were constructed herein, and their target RNA specificity, transgene controllability, tissue selectivity, liver toxicity, and anti-cancer efficacy were analyzed in mouse models orthotopically implanted with not only xenografts but also syngeneic HCC.

## Results

### Selective induction of cytotoxicity in human liver cancer cells by miR-122a-regulated *trans*-splicing ribozyme

**Figure 2:**
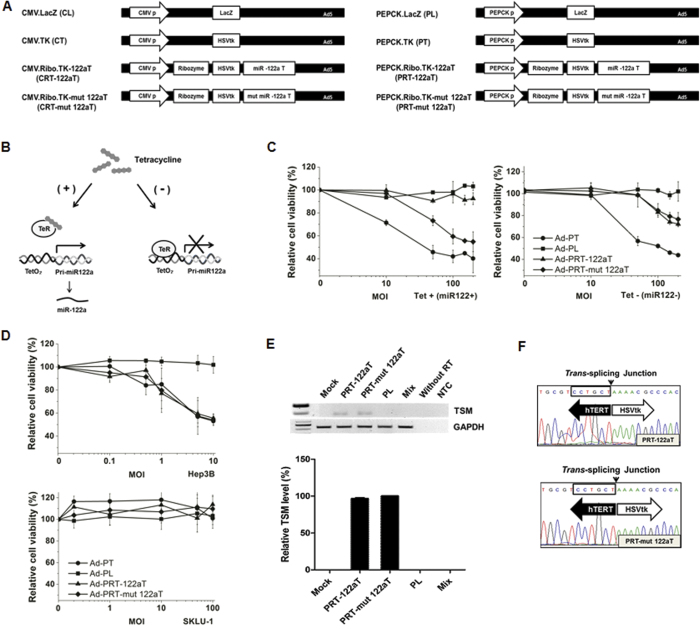
MiR-122a-regulated hTERT-specific cytotoxicity of Ad-PRT-122aT. (**A**) Expression vectors encoding hTERT-targeting ribozymes. (**B**) Schematic diagram of the tetracycline-inducible system for miR-122a expression in HepG2 cells. (**C**) Selective cytotoxicity of the ribozyme-encoding adenoviral vectors according to miR-122a. Stable HepG2-Tet-on cells expressing miR-122a were infected with each adenoviral vector at various MOI and inoculated with 100 μM GCV (Left panel; with 100 μM tetracycline, right panel; without tetracycline). (**D**) Hep3B or SKLU-1 cells were infected with each adenovirus at various MOI and inoculated with 100 μM GCV. Cell viability was determined via MTS assay. Results were presented as means ± SD of triplicate experiments. (**E**) RNA analysis of the adenoviral-infected cells. The Hep3B cells were mock-infected (Mock) or infected with PEPCK-ribozyme adenovirus at 10 MOI. SKLU-1 cells infected with Ad-PRT-122aT were mixed with mock-infected Hep3B cells (mix). TSMs generated in the cells were amplified, yielding a DNA fragment of 187 bp. Human GAPDH RNA was amplified as an internal control. NTC denotes non-template control. Note that cropped gel images are used in this figure and the gels were run under the same experimental conditions (upper panel). RNA level of TSM was measured using qRT-PCR in Hep3B cells infected with each adenovirus and expressed as a percentage of TSM level of Ad-PRT-mut 122aT. Data are mean values ± SD of triplicate experiments (bottom panel). (**F**) Representative sequences of TSMs generated from Hep3B cells infected with Ad-PRT-122aT or Ad-PRT-mut 122aT in (**E**) were shown.

We first evaluated the availability of miR-122a target sites (122aT) for the selective transgene regulation (Supplementary Fig. 1). Next, expression vectors encoding hTERT-targeting ribozymes harboring the suicide gene (herpes simplex virus thymidine kinase, HSVtk) as the 3′ exon and 122aT in the 3′-UTR under the control of cytomegalovirus (CMV) or liver-specific phosphoenolpyruvatecarboxykinase (PEPCK) promoter (CRT-122aT and PRT-122aT, respectively) were constructed to develop *trans*-splicing ribozymes with the ability to selectively trigger therapeutic transgene activity in hTERT(+) cancer cells, but not in normal liver cells (Fig. 2A). The PEPCK promoter was the most effective in liver cancer cells among the liver-specific promoters^13^. For comparison and target site controls, hTERT-specific ribozymes with mutant miR-122a target sites were constructed (CRT-mut 122aT and PRT-mut 122aT). Expression vectors encoding the β-galactosidase (LacZ) gene (CL and PL) were also constructed as negative controls, along with vectors encoding the HSVtk gene (CT and PT) as positive controls. The miR-122a-regulated ribozymes selectively stimulated therapeutic HSVtk expression and thus selectively and efficiently conferred prodrug ganciclovir (GCV) susceptibility to hTERT(+) liver cancer cells lacking miR-122a, but not to hTERT(+) and miR-122a(+) Huh7 cells (Supplementary Fig. 2).

To evaluate the anticancer effects and miR-122a dependency *in vivo*, recombinant adenoviral vectors encoding for the *trans*-splicing ribozymes, target site controls, and positive and negative controls under the control of the CMV (Ad-CRT-122aT, Ad-CRT-mut 122aT, Ad-CT, and Ad-CL) or PEPCK promoter (Ad-PRT-122aT, Ad-PRT-mut 122aT, Ad-PT, and Ad-PL) were generated. Liver cancer HepG2 cell lines stably expressing pri-miR-122a tetracycline-dependently were established to determine whether the cytotoxicity of adenoviral vectors encoding the ribozyme is regulated by miR-122a (Fig. 2B). This cell system could be more advantageous to study the ribozyme controllability than inherently different cell lines because of homogenous viral infectivity and promoter activity. Ad-PRT-mut 122aT and Ad-PT stimulated cytotoxicity regardless of the miR-122a expression in the cells (Fig. 2C). In contrast, Ad-PRT-122aT selectively induced HSVtk gene activity in an MOI-dependent manner in stable cells not treated with tetracycline (miR-122a negative) similarly to Ad-PRT-mut 122aT, but not in the tetracycline-treated cells (miR-122a positive).

Ad-CT was cytotoxic in cell lines independent of hTERT status, whereas Ad-CRT-122aT and Ad-CRT-mut 122aT specifically killed hTERT(+) HCC cells, but not hTERT(–) SKLU-1 cells, indicating target RNA specificity of the ribozyme (Supplementary Fig. 3A). In addition, Ad-PT, Ad-PRT-122aT, and Ad-PRT-mut 122aT also killed hTERT(+) Hep3B cells, but not SKLU-1 cells (Fig. 2D). Notably, the cytotoxic activities of Ad-PRT-122aT and Ad-PRT mut122aT were equivalent to that of Ad-PT in Hep3B cells. Infection with Ad-CRT-122aT and Ad-CRT-mut 122aT, or Ad-PRT-122aT and Ad-PRT-mut 122aT generated *trans*-spliced molecules (TSMs) in HCC cells through precise targeting U21 of the hTERT RNA (Supplementary Fig. 3B, and Fig. 2E,F). No difference in the mature miR-122a levels were observed between Ad-PRT-122aT- and Ad-PRT-mut 122aT-infected Huh7 cells during infection (Supplementary Fig. 4). However, Ad-CRT-122aT infection induced the expression of the cyclin G1 gene, one of the miR-122a target genes^27^, in Huh7 cells (data not shown), probably due to sequestering of the endogenous miR-122a by high expression of miR-122aT. Therefore, Ad-PRT-122aT was further featured as a therapeutic reagent against HCC.

### Efficient regression of HCC xenografts, diminished hepatotoxicity, and miR-122a regulation *in vivo* by the systemic delivery of the ribozyme-encoding adenovirus

**Figure 3:**
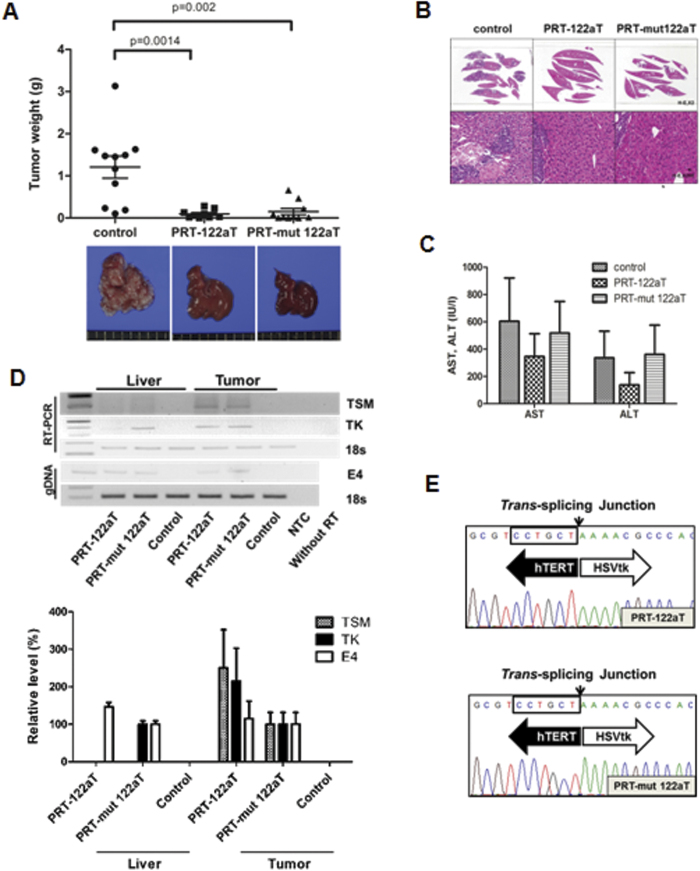
Anti-cancer efficacy and regulation of transgene expression of the adenovirus in xenograft model of HCC. (**A**) Ten mice with multifocal HCC per group were systemically administered with 1 × 10^11^ v.p. of each adenoviral vector, followed by GCV treatment. The HCC weights of each mice group were determined and plotted. Average tumor mass was presented with SD. Representative livers with tumor burdens of each mouse group treated with GCV were photographed (bottom panel). (**B**) Microscopic findings of sliced entire livers from each representative mouse in (**A**). The sectioned and paraffin-embedded liver tissues were stained with H&E. Microscopically, deep blue-colored nodules indicate HCC, while red-colored tissues are non-tumoral liver cells. (**C**) Liver enzyme levels in the virus and GCV-treated mice were represented as means ± SD. (**D**) RNA and genomic DNA patterns of mice injected with adenovirus. Ribozyme, transgene expression (HSVtk, TK), or TSM production was analyzed using RT-PCR. Endogenous 18S RNA was amplified as an internal control. Adenovirus genome (E4) and 18S DNA were analyzed using genomic DNA PCR. NTC denotes non-template control. Note that cropped gel images are used in this figure and the gels were run under the same experimental conditions (upper panel). TSM or TK RNA level and adenovirus genome level was measured using real-time PCR in liver or tumor tissues from mice infected with each adenovirus and expressed as a percentage of the level of Ad-PRT-mut 122aT. Data are average values ± SD (bottom panel). (**E**) Representative sequences of TSMs generated from tumor in the HCC mouse model infected with Ad-PRT-122aT or Ad-PRT-mut 122aT in (**D**) were shown.

Systemic delivery of Ad-PRT-122aT combined with the administration of GCV was well tolerated with minimal liver toxicity in normal C57BL mice (Supplementary Fig. 5). Antitumor efficacy was examined after systemic delivery of each adenoviral vector with GCV treatment into orthotopic and multifocal HCC mouse models established by splenic subcapsular inoculation of hTERT(+) Hep3B cells in BALB/c nude mice (Fig. 3). No mice in the Ad-PT group survived due to liver failure. The mean values of the tumor mass (g) were as follows: 1.21 ± 0.87 for the control/GCV group, 0.10 ± 0.10 for the Ad-PRT-122aT/GCV group, and 0.15 ± 0.23 for the Ad-PRT-mut 122aT/GCV group. Significant reductions in tumor generation were noted in the group of mice treated with both Ad-PRT-122aT and Ad-PRT-mut 122aT as compared with the control group (ANOVA; *p *< 0.002; Fig. 3A). While the livers of the control group were mostly replaced by multiple tumor nodules, the livers of the Ad-PRT-122aT and Ad-PRT-mut 122aT infected groups revealed no apparent tumor nodules, grossly (Fig. 3A bottom), or only tiny detectable tumor nodules, microscopically (Fig. 3B). The least toxicity was observed in liver treated with Ad-PRT-122aT, compared with Ad-PRT-mut 122aT and the control group, in the tumor xenografted mice (Fig. 3C). TSM was generated in the tumors of both mice infected with Ad-PRT-122aT and Ad-PRT-mut 122aT through accurate targeting of the hTERT RNA (Fig. 3D,E). Notably, the level of ribozyme expression was remarkably lower in normal livers treated with Ad-PRT-122aT than Ad-PRT-mut 122aT (Fig. 3D).

### Selectively regulated induction of cytotoxicity by mTERT-targeting *trans*-splicing ribozyme in mouse liver cancer cells

**Figure 4:**
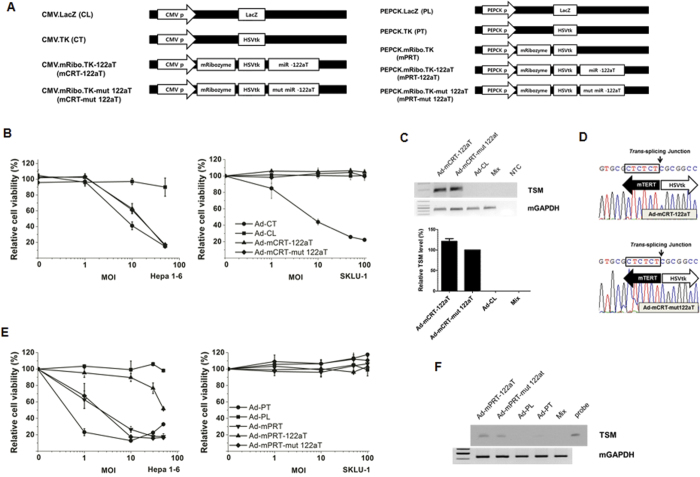
mTERT-targeted HSVtk RNA expression and cytotoxicity through miR-122a control in cells. (**A**) Schematic diagram of adenoviral backbone vectors. (**B**) Hepa 1–6 or SKLU-1 cells were infected with each adenovirus and inoculated with 100 μM GCV. Cell viability was determined via MTS assay. Results represent the means ± SD of three independent experiments. (**C**) RNA analysis of the adenovirus-infected cells. Hepa 1–6 cells were infected with each adenovirus at 10 MOI. SKLU-1 cells infected with Ad-mCRT-122aT were mixed with mock-infected Hepa 1–6 cells (mix). TSMs generated in the cells were amplified, yielding a DNA fragment of 177 bp. Mouse GAPDH RNA was amplified as an internal control (upper panel). RNA level of TSM was measured using qRT-PCR in Hepa 1–6 cells infected with each adenovirus and expressed as a percentage of TSM level of Ad-mCRT-mut 122aT. Data are mean values ± SD of triplicate experiments (bottom panel). (**D**) Representative sequences of TSMs generated from the cells infected with Ad-mCRT-122aT or Ad-mCRT-mut 122aT in (**C**) were shown. (**E**) Cytotoxicity of each adenovirus and GCV treatment in Hepa 1–6 and SKLU-1 cells. (**F**) TSMs of Ad-mPRT-122aT and Ad-mPRT-mut 122aT in Hepa 1-6 cells were detected by southern blotting. Note that cropped gel images are used in this figure and the gels were run under the same experimental conditions.

To validate the specificity and efficacy of the TERT-targeting *trans*-splicing ribozyme in a syngeneic orthotopic murine model of HCC, adenovirus encoding a mouse TERT (mTERT)-targeting *trans*-splicing ribozyme (Ad-mCRT) was constructed, and the transgene expression and stimulation of cytotoxic activity in mTERT(+) cells by the adenovirus was confirmed (Supplementary Fig. 6). Normal tissues in mouse, including the liver, express much higher levels of telomerase compared with human tissues^28^, indicating that mouse with syngeneic HCC would provide a system to strictly test the specificity of TERT-targeting anti-cancer approaches. To develop a *trans*-splicing ribozyme that can selectively trigger therapeutic transgene activity in mTERT(+) cancer cells but not in normal mouse liver cells, an expression vector encoding the mTERT-specific ribozyme with the HSVtk gene as the 3′ exon and the 122aT sequence in the 3′-UTR (mCRT-122aT) was constructed (Fig. 4A). Transgene expression and cytotoxicity were selectively down-regulated in mCRT-122aT transfected Hepa 1–6 cells by pre-miR-122a but not by mutant pre-miR-122a (Supplementary Fig. 7).

Moreover, the recombinant adenoviruses, Ad-mCRT-122aT and Ad-mPRT-122aT, were constructed for *in vivo* use (Fig. 4A). Ad-CT was cytotoxic in cells independent of the mTERT status, whereas Ad-mCRT-122aT and Ad-mCRT-mut 122aT specifically killed mTERT(+) Hepa 1–6 cells but not mTERT(-) SKLU-1 cells, indicating target RNA specificity of the ribozyme (Fig. 4B). TSM was generated in both Ad-mCRT-122aT- and Ad-mCRT-mut 122aT-infected Hepa 1–6 cells through accurate targeting of the mTERT RNA (Fig. 4C,D). Furthermore, Ad-mPRT and Ad-mPRT-mut 122aT also killed Hepa 1–6 cells but not SKLU-1 cells (Fig. 4E). Notably, the specific cytotoxicity of Ad-mPRT-122aT was observed, but with much less efficacy in Hepa 1–6 cells, probably because of the expression of small amounts of miR-122a in those cells (data not shown). TSMs were observed in the cells infected with both Ad-mPRT-122aT and Ad-mPRT-mut 122aT (Fig. 4F).

### MiR-122a-mediated selective regulation and efficient regression of HCC by Ad-mPRT-122aT with minimal hepatotoxicity in syngeneic orthotopic mouse model

**Figure 5:**
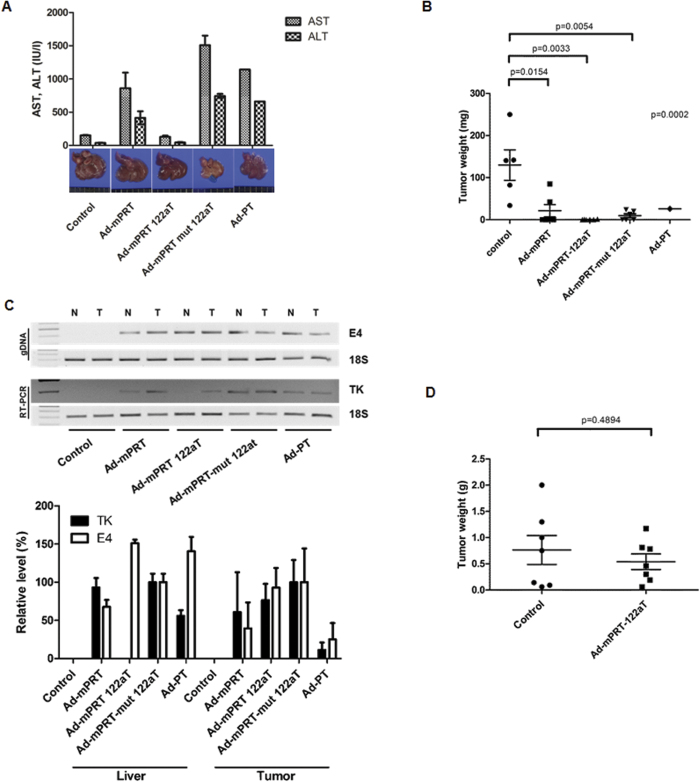
Cancer-specific regression by miR-122a-regulated ribozyme and GCV in syngeneic orthotopic model of HCC. (**A**) Syngeneic orthotopic C57BL mice with multifocal HCC were systemically administered with 1 × 10^11^ v.p. of each adenoviral vector (*n* = 7 each), followed by GCV treatment. Liver enzyme levels of the mice were measured and represented as means ± SD. A representative liver with tumor burdens of each group was photographed (bottom panel). (**B**) The HCC weights of each group in (**A**) were determined and plotted. Average tumor mass was presented with SD. (**C**) Ribozyme RNA (HSVtk, TK) and viral genomic DNA (E4) patterns of normal liver tissue (lane N) and HCC (lane T) from syngeneic mice model infected with adenovirus were analyzed using PCR. The 18S RNA and 18S genomic DNA were amplified as internal controls. Note that cropped gel images are used in this figure and the gels were run under the same experimental conditions (upper panel). TK RNA level and virus genomic DNA level was quantified using real-time PCR in liver or tumor tissues from mice infected with each adenovirus and expressed as a percentage of the level of Ad-mPRT-mut 122aT. Data are average values ± SD (bottom panel). (**D**) Seven mice of the allogenic and athymic BALB/c model with orthotopic and multifocal HCC for each group were systemically administered with 1 × 10^11^ v.p. of adenoviral vectors and GCV. The HCC weights of each group were determined and plotted. Average tumor mass was presented with SD.

Systemic delivery of Ad-mPRT-122aT was well tolerated with least liver toxicity when inoculated with GCV in normal C57BL mice and resulted in the down-regulation of ribozyme expression but no effects on the endogeneous miR-122a level in the livers of the mice (Supplementary Fig. 8). MiR-122a regulation and antitumor efficacy was examined after systemic delivery of each adenoviral vector with GCV treatment into syngeneic orthotopic and multifocal HCC mouse models established by splenic subcapsular inoculation of Hepa 1–6 cells into C57BL mice. The liver enzyme levels of the Ad-mPRT/GCV and Ad-mPRT-mut 122aT/GCV groups were highly increased in the same manner as the Ad-PT/GCV group, likely because of mTERT expression in mouse normal liver tissue (Fig. 5A). Notably, only one mouse in the Ad-PT/GCV group survived due to disseminated induction of suicide gene activity. In sharp contrast, minimal liver toxicity was observed in the Ad-mPRT-122aT/GCV infected mice. Moreover, the number of tumor nodules and their volume was significantly regressed in mice administered with Ad-mPRT-122aT/GCV compared with the control mice (Fig. 5A bottom and 5B). Of note, the Ad-mPRT-122aT-treated livers revealed only the least microscopically detectable tumor nodules. Mice with Ad-mPRT/GCV, Ad-mPRT-mut 122aT/GCV, and Ad-PT/GCV also showed efficient retardation of tumor nodules and volume. However, the non-tumoral livers of mice in these groups were abnormal and bleached, due to liver toxicity. Transgene was expressed both in non-tumoral liver tissue and in HCC samples from mice infected with Ad-mPRT, Ad-mPRT-mut 122aT, or Ad-PT. In contrast, ribozyme RNA was generated only in the HCC sample, not in the non-tumoral liver in Ad-mPRT-122aT-treated mice (Fig. 5C).

To monitor the mechanism by which Ad-mPRT-122aT caused efficient regression of HCC, an allogenic and athymic mouse model of orthotopic HCC was generated by splenic subcapsular inoculation of Hepa 1–6 cells in BALB/c nude mice and the antitumor efficacy was examined after systemic delivery of Ad-mPRT-122aT or control with GCV (Fig. 5D). The mean values of the tumor masses (g) were as follows: 0.76 ± 0.73 for the control/GCV group and 0.54 ± 0.40 for the Ad-mPRT-122aT/GCV group. About 29% reduction in the tumor volume was observed in the group of mice infected with Ad-mPRT-122aT, but this reduction was statistically insignificant.

### Acquisition of tumor immunity in syngeneic orthotopic mouse model of HCC treated with Ad-mPRT-122aT and GCV

**Figure 6:**
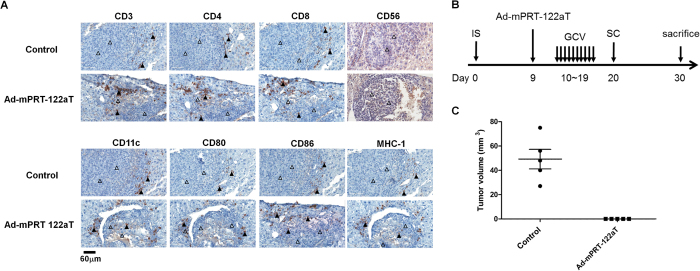
Induction of systemic anti-tumor immunity by treatment with Ad-mPRT-122aT and GCV. (**A**) Immune cell infiltration during *in vivo* assessment of antitumor effects of Ad-mPRT-122aT. The liver tissue of syngeneic HCC mice treated with Ad-mPRT/GCV or control/GCV was stained with each antibody. Empty triangles indicate cancer cells and filled triangles represent immunohistochemically stained cells. Scale bar was shown at bottom left corner. (**B**) Schematic representation of challenge experiments with parental HCC after adenovirus infection and GCV treatment *in vivo*. Syngeneic mice (*n *= 5) were given intrasplenic injection of Hepa 1–6 cells and infected with 1 × 10^11^ v.p. of virus on day 9. GCV treatment was given from days 10 to 19, and 6 × 10^6^ Hepa 1–6 cells were subcutaneously injected into the flanks of the mice on day 20. Tumors were harvested on day 30. (**C**) Tumor volumes of subcutaneous HCC mass of each group of mice were determined and plotted. Average tumor volume was presented with SD.

Immmunohistochemistry analysis of the liver tissues of Ad-mPRT-122aT/GCV treated syngeneic and immune-competent C57BL mouse with orthotopic HCC showed that CD3(+), CD4(+), CD8(+), CD11c(+), CD80(+), CD86(+), and MHC-1(+) cells, but not CD56(+) cells, were significantly infiltrated in the tumor microenvironment (Fig. 6A). However, infiltrations of the cells were shown at the boundary between the tumor and surrounding liver tissue in the control group.

To examine whether the ribozyme can systemically trigger immunity to HCC, syngeneic mice given orthotopic HCC through splenic subcapsular injection of the liver cancer cells were treated with the ribozyme-encoding virus or control and GCV and then re-challenged with the parental tumor cells at distant sites by lateral subcutaneous injection of Hepa 1–6 cells (Fig. 6B). The livers of the control group were mostly replaced by multiple tumor nodules (Supplementary Fig. 9). In contrast, the livers of the Ad-mPRT-122aT infected group revealed no apparent tumor formation with negligibly detectable tumor nodules observed microscopically. Moreover, lateral tumor nodules were efficiently formed on the challenged control mice group, whereas no tumors were generated in the Ad-mPRT-122aT-treated group (Fig. 6C).

## Discussion

The shortcomings of anti-cancer agents which target telomerase involve the lag phase between the time of telomerase inhibition and the time of telomere shortage for conferring damage to cancer growth, or the possibility of increase in the genomic stability of the surviving cancer cells^29^. In contrast, the approach with a *trans*-splicing ribozyme will be more advantageous because of the cumulative effects of reducing the level of hTERT and concurrent targeted stimulation of therapeutic gene activity^14^,^15^. However, one concern regarding telomerase-targeting is its imperfect selectivity for cancer, causing the possibility of targeting normal telomerase-expressing cells such as hematopoietic cells, germ cells, and regenerating normal cells.

Adopting a liver-specific promoter for the ribozyme system enabled the therapeutic activity to be more selective to HCC^16^. However, most HCC patients are diagnosed at late stages when only few normal hTERT(+) regenerating liver cells would remain. Therefore, the use of a liver-specific promoter alone to express the ribozyme could not completely avoid the possibility of liver failure due to targeting of the normal liver in such patients. Herein, expression of the ribozyme was controlled to strictly occur in TERT(+) HCC cells, in which miR-122a levels are highly repressed, by inserting antisense sequences against liver-specific miR-122a into the ribozyme cassette. Our results in syngeneic as well as xenografts animal models orthotopically implanted with multifocal HCC demonstrated that functional HSVtk protein by the miRNA-regulated ribozyme was selectively expressed in HCC, but not in the normal liver tissue, resulting in efficient tumor regression with the least liver toxicity. Notably, minimal hepatotoxicity was observed in the syngeneic HCC mice even if they expressed high levels of mTERT(+) in the liver when adenovirus encoding the ribozyme was systemically delivered, followed by GCV treatment, indicating strict selective targeting of the HCC by the ribozyme.

Cancer-specific targeting through cell- or condition-specific induction of therapeutic transgene has been proposed as an anticancer approach. Recently, cancer-specific induction of transgene activity through miRNA regulation was described as one of the cancer-targeting strategies^22^,^30^. Although the approaches improve cancer-specific targeting compared to expression of therapeutic gene alone, nonspecific expression of the gene can still occur in cells other than cancers which do not express the miRNA. Contrarily, herein we combined two post-transcriptional targeting approaches, miRNA regulation and an RNA replacement strategy, to induce the expression of therapeutic genes more strictly in cancer cells. Targeting too many endogenous miRNAs through multiple copies of the miRNA targets or overexpression of the target site-harboring transgene construct could cause potential adverse effects by acting as a miRNA sponge^31^. In this study, we developed a PEPCK promoter-driven ribozyme construct with three copies of 122aT without causing any effect on the intracellular miR-122a level, indicating that the expression level and copy number of 122aT in our expression cassette would not cause a sponge effect and adverse effects to the endogenous miRNA. The endogenous miR-122a level will also determine the degree of transgene inhibition in the normal liver without sequestering the miRNA^22^.

With regard to anticancer efficacy, the miR-122a-regulated ribozyme was evidently as potent as the unregulated ribozyme, with the least hepatotoxicity, in both xenografts and syngeneic animal models orthotopically implanted with multifocal HCC when systemically administered and followed by GCV treatment. In the xenograft model implanted with hTERT(+) HCC, no significant differences in the efficacy were observed between the miRNA-regulated (Ad-PRT-122aT) and unregulated ribozymes (Ad-PRT-mut 122aT), likely due to no change in ribozyme expression level by inserting 122aT into the ribozyme expression cassette in HCC. In other words, incorporating target sequences to specific miRNA into the ribozyme enhanced the cancer-targeting selectivity without compromising its anticancer efficacy. Moreover, the observation of the anti-cancer effect in the xenografts of athymic mice treated with NK cell inhibitor suggests that the anticancer activity in the animal induced by Ad-PRT-122aT/GCV was mostly due to suicide gene activation with bystander effects^32^ through the precise RNA replacement with hTERT RNA.

In contrast, no obvious anti-cancer phenomena were shown in the allogenic and NK inhibitor-treated immune-deficient mice with orthoptopic mouse HCC when exposed to the miR-122a-regulated mTERT-targeting ribozyme (Ad-mPRT-122aT) and GCV treatment. This result is most likely due to more than 40-fold inefficient *trans*-splicing activity of the mTERT-targeting ribozyme compared with the hTERT-targeting ribozyme^13^,^33^. Moreover, low levels of endogenous miR-122a in the implanted Hepa 1–6 cells could cause inefficient ribozyme activity, as shown in the MTS experiments. However, the observation of significant tumor regression in immunologically competent syngeneic mice with orthotopic HCC after Ad-mPRT-122aT/GCV treatment indicates that acquired immunity from the lysed and apototic cancer cells due to suicide gene activity would be a major factor causing the selective and effective anti-cancer effects of the mTERT-targeting ribozyme. Apoptosis of cancer cells caused by HSVtk/GCV was reported to induce cancer-specific immunity likely due to tumor-infiltrating dendritic cells in mice^34^, suggesting that minor RNA replacement activity of the mTERT-specific ribozyme could induce immunogenic cell death. Accordingly, our immunhistochemistry experiments showed that dendritic cells and activated CD8(+) T cells, but not NK cells, were infiltrated into the tumor environments in the syngeneic mouse treated with Ad-mPRT-122aT/GCV. Moreover, growth of the secondary challenged parental tumors at distal sites in the mice was completely suppressed. Taken together, the *in vivo* results suggest that mPRT-122aT has significant and specific anti-HCC efficacy through HCC-specific *trans*-splicing reactions modulated by the liver-specific miR-122a and local and systemic induction of specific immunity to the cancer cells with prodrug inoculation.

In summary, we developed a new HCC gene therapeutic strategy with high cancer-selectivity, the least targeting of normal cells, and efficient anticancer effects, based on the targeted replacement of TERT RNA through a *trans*-splicing ribozyme under control of liver-specific miR-122a expression. We evidenced the anti-HCC effects by the TERT-specific ribozyme not only in orthotopically implanted human HCC xenografts but also in a syngeneic immunocompetent HCC mouse model. TERT expression, although variable among HCC, is activated in the majority of HCCs^35^. The expression of miR-122a is relatively high in well differentiated tumors, but specifically repressed in poorly differentiated HCCs^36^. Therefore, a subgroup of HCC such as tumors with a bad prognosis signature and aggressive phenotype, which is difficult to be handled by current therapeutic approaches, could most likely benefit from our developed strategy. *Trans*-splicing ribozymes targeting cancer-associated transcripts other than TERT RNA are now being developed with *in vivo* efficacy and specificity^37^,^38^. MiRNAs specifically related with a broad range of human cancers have been intensively unearthed^39^. Together with these advances, a cancer-specific targeted RNA replacement strategy controlled by miRNA expression could provide effective therapeutic options to diverse human cancers.

## Methods

### Design of ribozyme

Expression vectors encoding for hTERT or mTERT-specific *trans*-splicing ribozymes under the control of CMV or PEPCK promoter were constructed via cloning the ribozyme construct into pcDNA or pPEPCK-LCR, as previously described^13^,^33^, with modification. The hTERT-targeting ribozyme was designed to be directed at the +21 uridine residue on hTERT RNA and contained an extended internal guide sequence, including an extension of the P1 helix, an additional 6 nt-long P10 helix, and a 325 nt-long antisense sequence against the downstream region (+30 to +354 residue) of the hTERT RNA targeted site. The mTERT-specific ribozyme targeted the +67 uridine site on mTERT RNA and harbored a sequence of extended P1 helix, additional 6 nt-long P10 helix, and a 100 nt-long antisense sequence complementary to the downstream region (+76 to +175 residue) of the targeted RNA site. HSV-TK cDNA was introduced as 3′ exon of the ribozymes. Three copies of the target site of miR-122a (122aT, 5′-ACAAACACCATTGTCACACTCCA-3′ × 3) or seed reverse sequence of miR-122a as a control (mut 122aT, 5′-ACAAACACCATTCCTCACACTGA-3′ ×3) were inserted into the 3′-UTR of the *trans*-splicing ribozyme expression constructs with *Bst*BI/*Not*I (constructs of CMV promoter) or *Bst*BI (constructs of PEPCK) site.

### Generation of recombinant adenoviral vectors

Expression vectors encoding the TERT-specific ribozyme with HSVtk gene at the 3′exon and three copies of 122aT at the 3′-UTR under the control of the CMV or PEPCK promoter were cloned into the pAdenoVator-CMV5-IRES-GFP shuttle vector (Qbiogene, Irvine, CA) to generate recombinant adenoviral vectors. Recombinant adenovirus vectors encoding the ribozymes were created by a homologous recombination technique in bacteria (BJ5183) as follows. In brief, the shuttle plasmid was linearized with *Pme*I and subsequently co-transformed into BJ5183 cells with an E1/E3 deleted adenoviral type5 backbone genome (pAdenoVator DE1/E3; Qbiogene). The recombinant vectors generated through homologous recombination in the bacteria were isolated and linearized with *Pac*I. The linearized vectors were then transfected into HEK293 cells, and the recombinant adenoviruses produced were isolated by three rounds of plaque purification. Recombinant viruses were amplified, purified, and concentrated via ultracentrifugation (Beckman-Coulter, Brea, CA). Titers of the recombinant adenovirus were determined by TCID_50_ analysis.

### Cells

Hepa 1–6 (murine hepatoma cell lines), Hep3B and HepG2 (human hepatoma cell lines), SKLU-1 (telomerase negative human lung carcinoma cell lines), and HEK293 (adenoviral E1-transformed human embryonic kidney cell lines) were purchased from the American Type Culture Collection (Manassas, VA). Huh7 (human hepatoma cell lines) was obtained from the Japanese Collection of Research Bioresources Cell Bank (Osaka, Japan). Hep3B and HepG2 were cultured in EMEM (Invitrogen, Carlsbad, CA) with 10% FBS. Hepa1–6, SKLU-1, Huh7, and HEK293 cells were maintained in DMEM (Invitrogen) with 10% FBS.

For the establishment of cell lines stably expressing miR-122a dependent on the presence of tetracycline, HepG2 cells expressing Tet repressor vector, pcDNA 6/TR (Invitrogen), were isolated with blasticidin for 2 weeks. The selected cells were transfected with vector encoding pri-miR-122a which was cloned into the tetracycline response element vector, pcDNA 4/myc-His B (Invitrogen), and zeocin resistant colonies were isolated for 2 weeks.

### MTS assay

Cells were seeded at 1 × 10^4^ cells per well in 96-well plates, incubated overnight and infected with varying multiplicities of infection (MOI) of the recombinant adenoviruses. At 1-day post-infection, 100 μM of GCV (CymeveneVR, Roche, Basel, Switzerland) was added to each plate. The media containing GCV as replaced every 48 h for 5 days, and CellTiter 96Aqueous one solution reagent (Promega, Madison, WI) in 100 μl of medium was added to each well and incubated for 1–4 h at 37 °C, based on the rate of color change. Cell viability was evaluated by determination of absorbance at 490 nm. Cell survivability after GCV treatment was quantitated as the fraction of the absorbance of cells without GCV treatment, and represented as the percentage relative to that of the uninfected cells.

### Nucleic acid analysis

To analyze the level of ribozyme RNA in adenoviral-infected cells or tissues from mice, total RNA was isolated with TriZol (Invitrogen) supplemented with 20 mM EDTA and reverse-transcribed with a specific (5′-AGTTAGCCTCCCCCATCTC-3′), oligo(dT), or random primer in the presence of 10 mM L-argininamide. The cDNAs were then amplified with HSVtk-specific primers (5′-TGACTTACTGGCAGGTGCTG-3′ and 5′-CCATTGTTATCTGGGCGCTTG-3′) through PCR.

For the *trans*-spliced RNA products in cells, mouse tissues, or tumors, the total RNA was reverse-transcribed with a primer specific for HSVtk (5′-AGTTAGCCTCCCCCATCTC-3′) in the presence of 10 mM L-argininamide, and the resulting cDNAs were amplified with a 5′ primer specific to the 5′ end of the mTERT (5′-GCCCATCCCGGCCTTGAG-3′) or hTERT sequence (5′-GGAATTCGCAGCGCTGCGTCCTGCT-3′) and a 3′ primer specific to the 3′ exon HSVtk sequence (5′-GTGAGGACCGTCTATATAAACCCGCAGTAG-3′). The amplified cDNA was then re-amplified with a 5′ nest primer specific for mTERT (5′-GTTGCCCCGCGGTGCGCTCT-3′) or hTERT (5′-GGAATTCGCAGCGCTGCGTCCTGCT-3′) and a nested 3′ primer specific to the HSVtk sequence (5′-CAGTAGCGTGGGCATTTTCT-3′). The products were then cloned and sequenced. In the case of Ad-mPRT-122aT and Ad-mPRT-mut 122aT, southern blotting was performed with the amplified cDNA from the adenovirus-infected cells using biotinylated DNA probe (5′-ATGACCCGCGCTCCTCGTTGCCCCGCGGTGCGCTCTCTGCGGCCG CGCTTCGTACCCCTGCCATCAAC-3′). For the internal control, cDNAs were amplified with 18S primers (5′-GTAACCCGTTGAACCCCATT-3′ and 5′-CCATCCAATCGGTAGTAGCG-3′) or GAPDH primers (5′-TGACATCAAGAAGGTGGTGA-3′ and 5′-TCCACCACCCTGTTGCTGTA-3′).

To detect the presence of adenoviruses in mice, DNA was isolated from various tissues and tumors with TriZol reagent, and the E4 gene of adenovirus was amplified. The primers used were 5′-GCTGGCCAAAACCTGCCCGCCGGCTATA-3′ and 5′-GCTCTAGATGGGTTGTTCCCTGGGATATGGTT-3′.

To quantify the levels of ribozyme RNA, *trans-spliced* RNA products, or adenoviral genomic DNA in cells, liver tissues, or tumors, reverse-transcribed HSVtk cDNA, *trans*-spliced cDNA, or E4 gene was amplified, respectively, through real-time PCR using SYBR-Green. For the ribozyme RNA level or adenovirus DNA amount, the threshold levels obtained from HSVtk or E4 PCR were adjusted to the threshold levels found in the reaction from 18S RNA or 18S genomic DNA, respectively, in order to correct for minor variations in DNA loading. For the *trans*-spliced RNA level, the obtained threshold levels were adjusted to those found in the GAPDH or 18S RNA reaction in order to normalize. Amplification was carried out in the Roter-Gene system, a real-time PCR machine (Corbett, San Francisco, CA).

### Animal studies

Male BALB/CAnN/CriBg-nu/nu nude mice or C57BL/6N mice (4 to 5 weeks old; NARA Bio, Seoul, Korea) were used. All procedures were authorized and approved by the Dong-A University College of Medicine ethics committee (project license No. DIACUC-13–28). The animals were kept under specific pathogen-free conditions and maintained in the Korean Food and Drug Administration animal facility in accordance with the AAALAC International Animal Care policy (accredited unit-Korea Food and Drug Administration: unit number: 000996). Nude mice were treated with 30 μl rabbit anti-asialo GM1 antibody (Wako Pure Chemical Industries, Osaka, Japan) 2 days prior to experimentation to inhibit NK cell activity.

A mouse model of multifocal and orthotopic HCC was established by splenic subcapsular inoculation of human HCC (Hep3B) or mice HCC (Hepa1–6) cells, as previously described^37^, with modification. A linear incision of the left flank abdominal wall was made to visualize the spleen, and 3 × 10^6^ cells in 100 μl of PBS were injected under the spleen capsule with a 29-gauge needle. The injection site was pressed with an aseptic cotton sponge for several minutes to prevent further leakage, after which the abdominal wall was sutured with silk. The mice showed multiple tiny tumor nodules along the liver margin, easily detectable by gross inspection, on the 11th or 12th day. On 12th day, 1 × 10^11^ v.p. of adenovirus encoding *trans*-splicing ribozyme, HSVtk gene without ribozyme, or LacZ or PBS as control were administered via tail vein, followed by intraperitoneal injection of GCV, 50 mg/kg, twice per day for 10 days from the day after virus injection. On the day following the last GCV treatment, blood sampling through the heart for liver enzyme (aspartate aminotransferase, ALT, or alanine aminotransferase, AST) analysis was carried out. All mice were euthanized, and the whole liver lobes were removed, measured, photographed, and serially sectioned in 2 mm thickness. Liver enzymes were measured by the Automated Blood Chemistry Line (Toshiba 200 FR, Tokyo, Japan). Entire liver slices from each mouse were fixed in 10% neutralized buffered formalin and processed for paraffin embedding. Tissue sections of 4- to 6-μm thickness were stained with hematoxylin and eosin (H&E) for morphologic examination. The microscopic images were scanned under virtual microscope (AperioTechnologica, Vista, CA). The tumor fraction was calculated using the AperioImagescope program v10.2.2.2319, and the tumor weight was estimated by multiplication of liver weight and tumor fraction.

### Immunohistochemistry

Tissue sections were placed on slides (Superfrost Plus microscope slide, Thermo Scientific, Braunschweig, Germany). Immunohistochemical staining was performed as follows using the DiscoveryXT automated immunohistochemistry stainer (Ventana Medical Systems, Inc., Tucson, AZ). Deparaffinization was carried out with EZ Prep solution (Ventana Medical Systems, Inc.). For antigen retrieval, CC1 standard [pH 8.4 buffer containing Tris/Borate/EDTA (Ventana Medical Systems, Inc.)] was used for 24 min. Treatment of Inhibitor D [3% H_2_O_2,_ endogenous peroxidase (Ventana Medical Systems, Inc.)] was carried out for 4 min at 37 °C. Slides were incubated with primary antibodies to anti-CD3-ε (Santa Cruz biotechnology, Inc., Santa Cruz, CA), CD4 (eBioscience, Inc., San Diego, CA), CD8 (Abbiotec, San Diego, CA), CD56 (Abcam, Cambridge, UK), CD11c (eBioscience, Inc.), CD80 (eBioscience, Inc.), CD86 (eBioscience, Inc.), and MHC Class I (eBioscience, Inc.) for 32 min at 37 °C. Treatment with secondary antibody, Dako REAL^TM^ Envision^TM^ anti-rabbit/mouse HRP (Dako, Glostrup, Denmark), was carried out for 16 min with anti-CD3-ε, anti-CD4, anti-CD56, anti-D11c, anti-CD80, anti-CD86, and anti-MHC Class I antibodies, and for 8 min with anti-CD8 antibody at 37 °C. Slides were incubated in DAB+ H_2_O_2_ substrate using the Ventana Chromo Map Kit (Ventana Medical Systems, Inc.) for 8 min followed by counterstaining with Hematoxylin and Bluing reagent.

### Statistical analyses

Statistical analysis was performed with Statistical Analysis System software (SAS Institute, Cary, NC). The differences between groups were assessed through analysis of variance (ANOVA). In the case of highly skewed distribution of measurements and small sample sizes, nonparametric statistical tests were used (Kruskal–Wallis test for overall comparison and Wilcoxon’s rank-sum test for pairwise comparison). All data were expressed as the average ± standard deviation (SD). *P*-values of <0.05 were considered statistically significant.

## Additional Information

**How to cite this article**: Kim, J. *et al.* Targeted Regression of Hepatocellular Carcinoma by Cancer-Specific RNA Replacement through MicroRNA Regulation. *Sci. Rep.* **5**, 12315; doi: 10.1038/srep12315 (2015).

## Supplementary Material

Supplementary Information

## Figures and Tables

**Figure 1:**
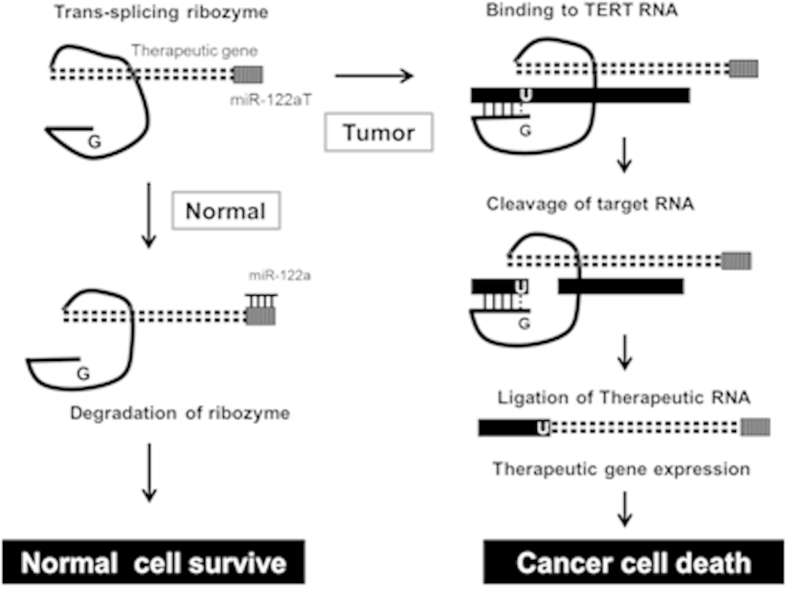
Scheme for the TERT-targeting *trans*-splicing ribozyme-induced selective expression of therapeutic RNA in cancer cells through microRNA regulation. *Trans*-splicing ribozymes with target sites to liver-specific miR-122a at the 3′-UTR of the 3′exon recognize TERT RNA at the targeted uridine residue by selective base-pairing through their internal guide sequence in cancer cells lacking the miRNA. The ribozymes then remove the sequence downstream of the target site and replace it with the 3′ exon exerting anti-cancer activity.
