# Supplementary material for: Targeted Regression of Hepatocellular Carcinoma by Cancer-Specific RNA Replacement through MicroRNA Regulation
Source: Sci Rep. 2015 Jul 20;5:12315. doi: 10.1038/srep12315 (PMC4507181; doi:10.1038/srep12315)
Supplement: Supplementary Information [file srep12315-s1.doc]

Supplementary Information:

Targeted Regression of Hepatocellular Carcinoma by Cancer-Specific RNA Replacement through MicroRNA Regulation

**Authors:** Ju Hyun Kim^1^, Ranhui Won^1^, Guyee Ban^1^, Mi Ha Ju^2^, Kyung Sook Cho^2^, Sang Young Han^3^, Jin-Sook Jeong^2^, Seong-Wook Lee^1^

**Affiliations:**

^1^Department of Molecular Biology, Institute of Nanosensor and Biotechnology, and Research Institute of Advanced Omics, Dankook University, Yongin 448-701, Republic of Korea.

^2^Department of Pathology and Immune-network Pioneer Research Center, ^3^Department of Internal Medicine, Dong-A University College of Medicine, Busan 602-714, Republic of Korea

Correspondence and requests for materials should be addressed to S.W.L. (email: [SWL0208@dankook.ac.kr](mailto:SWL0208@dankook.ac.kr)) or J.S.J. (email: [jsjung1@dau.ac.kr](mailto:jsjung1@dau.ac.kr))

**A**

**
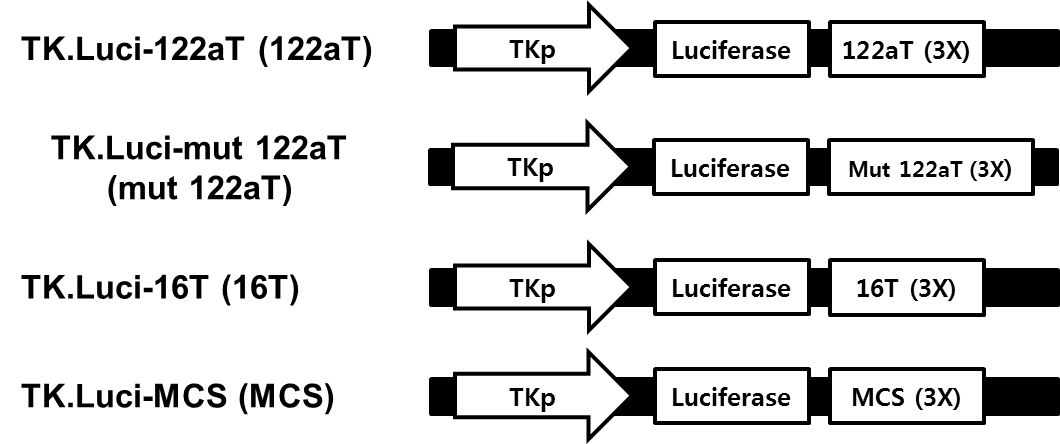
**  **
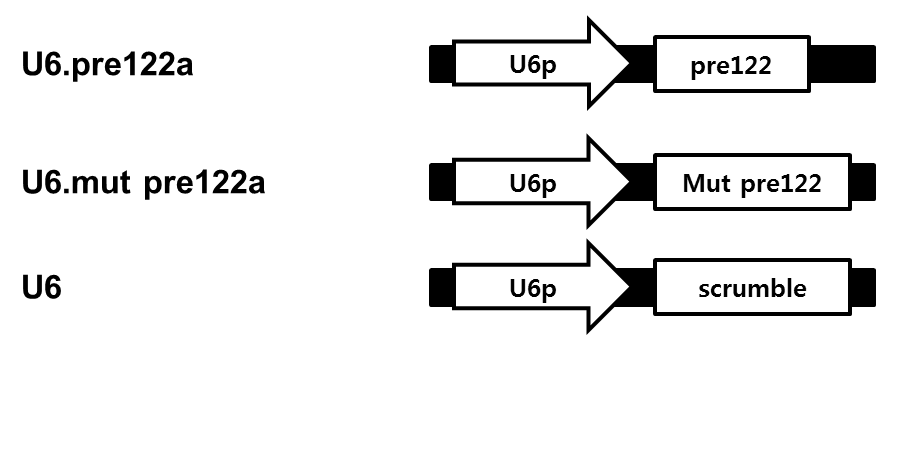
**

**B**

**
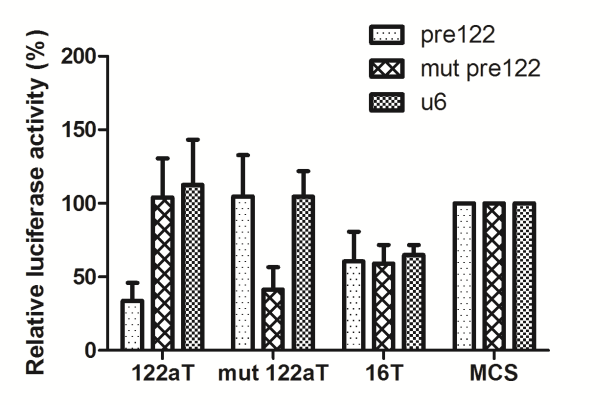
**

**Supplementary Figure 1. MiR-122a target sites for the selective regulation of transgene expression.** (A) Schematic diagram of constructs for reporter assay (B) MiR-122a-dependent regulation of transgene activity. HepG2 cells (miR-122a negative) were co-transfected with Renilla luciferase expression vector harboring miR-122a target sites (122aT), mutant miR-122a target sites (mut 122aT), miR-16 target sites (16T), or multicloning sites (MCS) as a control with firefly luciferase vector for an internal control and vector encoding pre-miR-122a, mutant pre-miR-122a or scrambled RNA under the U6 promoter. Luciferase activity was quantified and expressed as a percentage of the sample transfected with control luciferase vector. Results represent the means ± SD of three independent experiments. Transgene activity of the reporter construct harboring 122aT was selectively down-regulated by the expression of pre-miR-122a.

**A B**


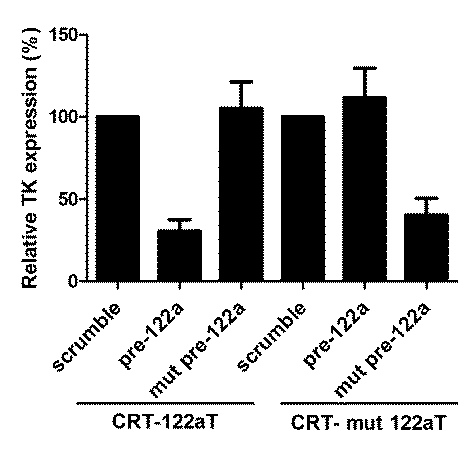

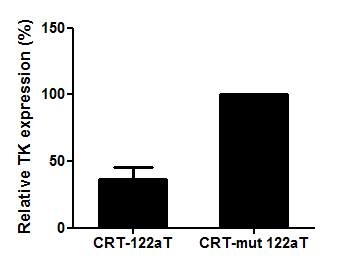


**C D**

**
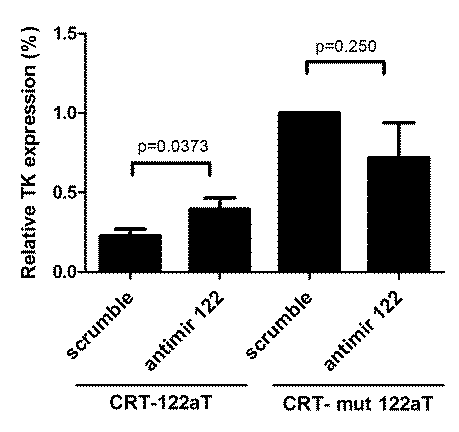
**  **
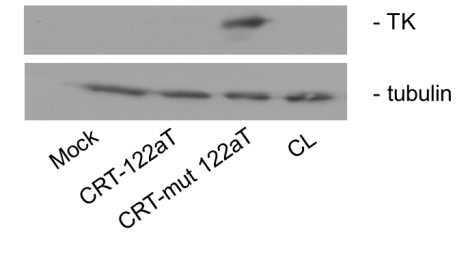
**

**E**


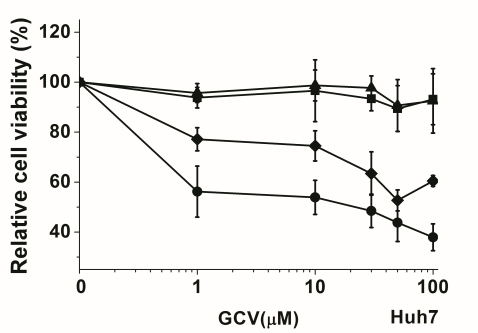

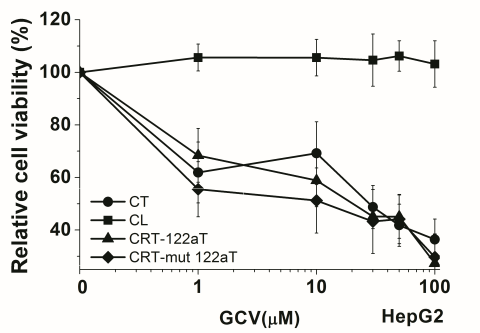


**F**

**
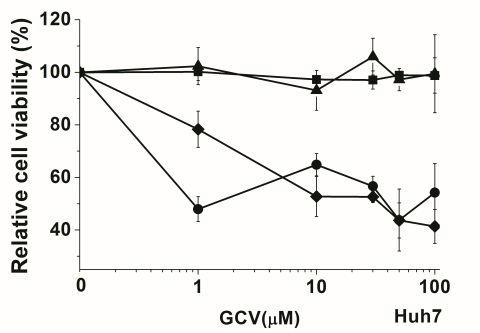
**  **
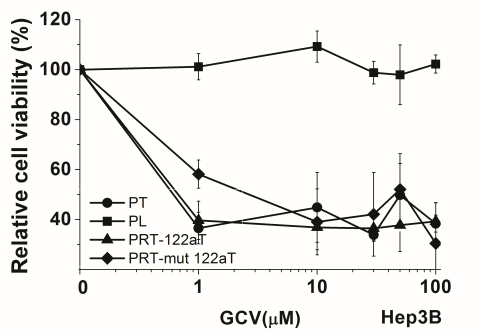
**

**Supplementary Figure 2. Selective HSVtk expression and cytotoxicity of hTERT-specific ribozymes under the control of miR-122a.** (A-C) Relative HSVtk RNA expression levels as assessed by qRT-PCR. The levels of ribozyme RNA in the ribozyme-transfected cells were quantified through amplification of HSVtk cDNA using real-time PCR. The threshold levels obtained from HSVtk were adjusted to the threshold levels found in the 18S reaction in order to correct for minor variations in cDNA loading. (A) Relative HSVtk RNA expression levels in Hep3B cells. Pre-miR-122a-encoding vector and CRT-122aT were co-transfected into Hep3B cells. CRT-mut 122aT was used as a control. The level of ribozyme transcripts (HSVtk RNA level) was selectively down-regulated in CRT-122aT transfected cells when co-transfected with pre-miR-122a in hTERT(+) and miR-122a(-) Hep3B cells. (B) Relative HSVtk RNA expression levels in Huh7 cells. The ribozyme RNA level was reduced by more than 60% in hTERT(+) and miR-122a(+) Huh7 cells transfected with CRT-122aT compared with cells transfected with CRT-mut 122aT. (C) Relative HSVtk RNA expression levels in Huh7 cells co-transfected with the ribozyme construct and antimir 122 RNA. The averages were represented with SD of three different transfected cells. Selective transgene regulation by miR-122a was confirmed with the transfection of antimir 122 RNA, which has the complementary sequence of mature miR-122a, in Huh7 cells. Antimir 122 rescued the level of ribozyme RNA transcripts that were down-regulated in the cells transfected with CRT-122aT, although the recovery by antimir 122 was partial, likely due to inefficient transfection. (D) HSVtk protein levels in ribozyme-transfected Huh7 cells were analyzed by western blotting. Tubulin was used as a loading control. For western analysis, proteins (30 μg) in soluble lysate were resolved by 10% SDS-PAGE and transferred to a polyvinylidenedifluoride membrane (Millipore, Billerica, MA). The membrane was blocked in 5% non-fat milk in PBS containing 0.1% Tween-20 (PBST) for 1 h at room temperature and then probed with antibodies specific to HSVtk (Santa Cruz Biotechnology) or tubulin (Santa Cruz Biotechnology). Subsequently, the membrane was thoroughly washed in PBST and incubated with HRP-conjugated secondary antibody for 1 h at room temperature. Finally, the membrane was developed by the enhanced chemiluminescence detection method (Amersham, Uppsala, Sweden). Note that cropped gel images are used in this figure and the gels were run under the same experimental conditions. The HSVtk protein level was decreased in Huh7 cells transfected with CRT-122aT, compared with the cells transfected with CRT-mut 122aT. (E) Efficacy and specificity of cancer cell killing activity of the miR-122a-regulated hTERT-targeting *trans-*splicing ribozyme. Each cell was transfected with CMV or (F) PEPCK promoter-driven ribozyme construct and inoculated with 1-100 μM GCV. Cell viability was determined via MTS assay. Values are expressed as the means ± SD of three independent experiments. Transfection of CRT-122aT into the Huh7 cells induced negligible cytotoxicity even at the highest concentrations of GCV, in contrast with the efficient induction of cytotoxicity observed for CRT-mut 122aT and CT in the cells. In sharp contrast, CRT-122aT rendered the HepG2 cells highly sensitive to GCV, with efficacy comparable to the effects by CRT-mut 122aT and CT. PRT-122aT also induced cytotoxicity in HepG2 cells when inoculated with GCV to a similar degree as observed by PT or PRT-mut 122aT, but not in Huh7 cells

**A**

**
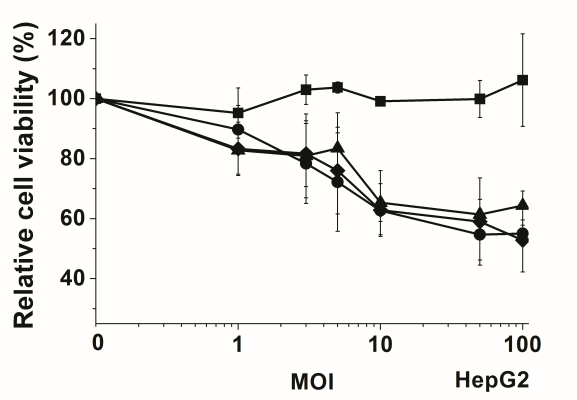
**  **
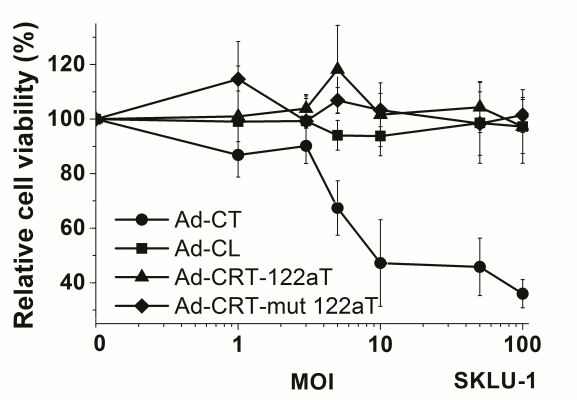
**

**B**

**
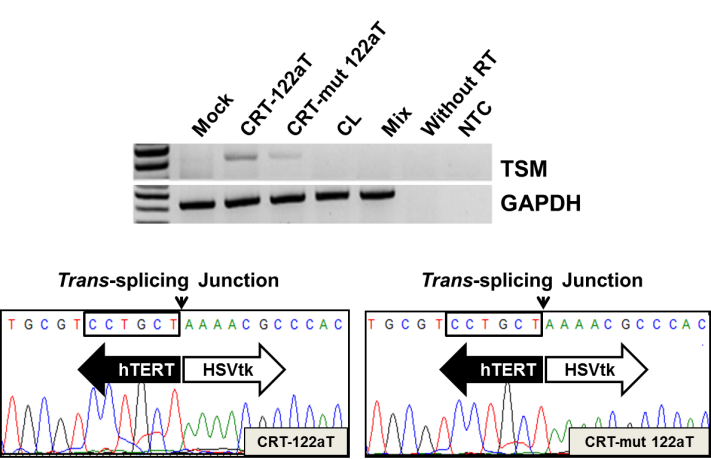
**

**Supplementary Figure 3. hTERT-dependent cytotoxicity induced by Ad-CRT-122aT.** (A) hTERT positive (HepG2) and negative (SKLU-1) cells were infected with Ad-CRT-122aT, Ad-CRT-mut 122aT, Ad-CT, or Ad-CL at various MOI and inoculated with 100 μM GCV. Cell viability was determined via MTS assay. Results were presented as means ± SD of triplicate experiments. (B) RNA analysis of adenovirus-infected cells. The hTERT(+) HepG2 cells were mock-infected (Mock) or infected with CMV-ribozyme adenovirus at 10 MOI. SKLU-1 cells infected with Ad-CRT-122aT were mixed with mock-infected HepG2 cells (mix). TSMs generated in the cells were amplified, yielding a DNA fragment of 187 bp. Human GAPDH RNA was amplified as an internal control. Note that cropped gel images are used in this figure and the gels were run under the same experimental conditions. No TSMs were detected in the RNA sample in the mix lane, indicating intracellular target RNA-specific *trans*-splicing reaction by the miR-122a-regulated ribozyme. Representative sequences of TSMs generated from HepG2 cells infected with Ad-CRT-122aT or Ad-CRT-mut 122aT were shown.

**
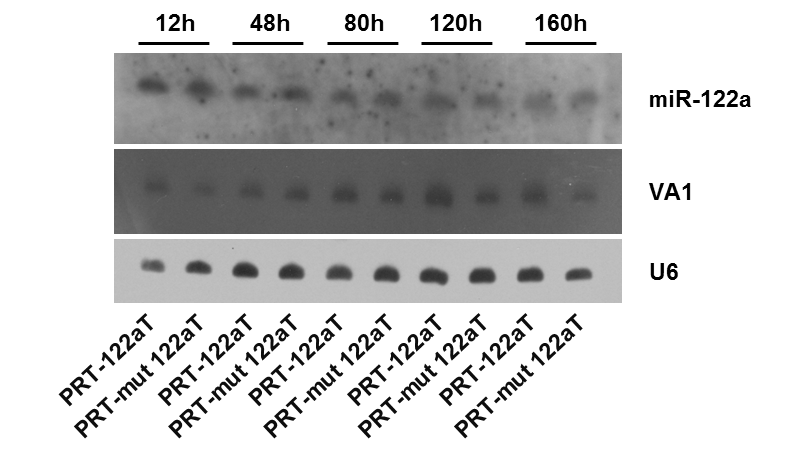
**

**Supplementary Figure 4. The level of mature miR122a in Huh7 cells after infection with Ad-PRT-122aT.** The levels of mature miR-122a were analyzed to test whether infection with Ad-PRT-122aT causes a sponge effect to miR-122a using northern blot assay at 12 h, 48 h, 80 h, 120 h, and 160 h post-infection with 10 MOI of Ad-PRT-122aT or Ad-PRT-mut 122aT. For northern analysis, RNA (10 μg) was resolved on a 12% urea-polyacrylamide gel and transferred electronically to a hyper charged-nylon membrane (Bio-Rad, Hercules, CA). An oligonucleotide complementary to miR-122a was end-labeled with biotin as the probe (5’-ACAAACACCATTGTCACACTCCA-3’). Adenoviral VA1 RNA and U6 RNA were detected as a virus infection control and an internal control, respectively. Note that cropped gel images are used in this figure and the gels were run under the same experimental conditions.

**A B**

**
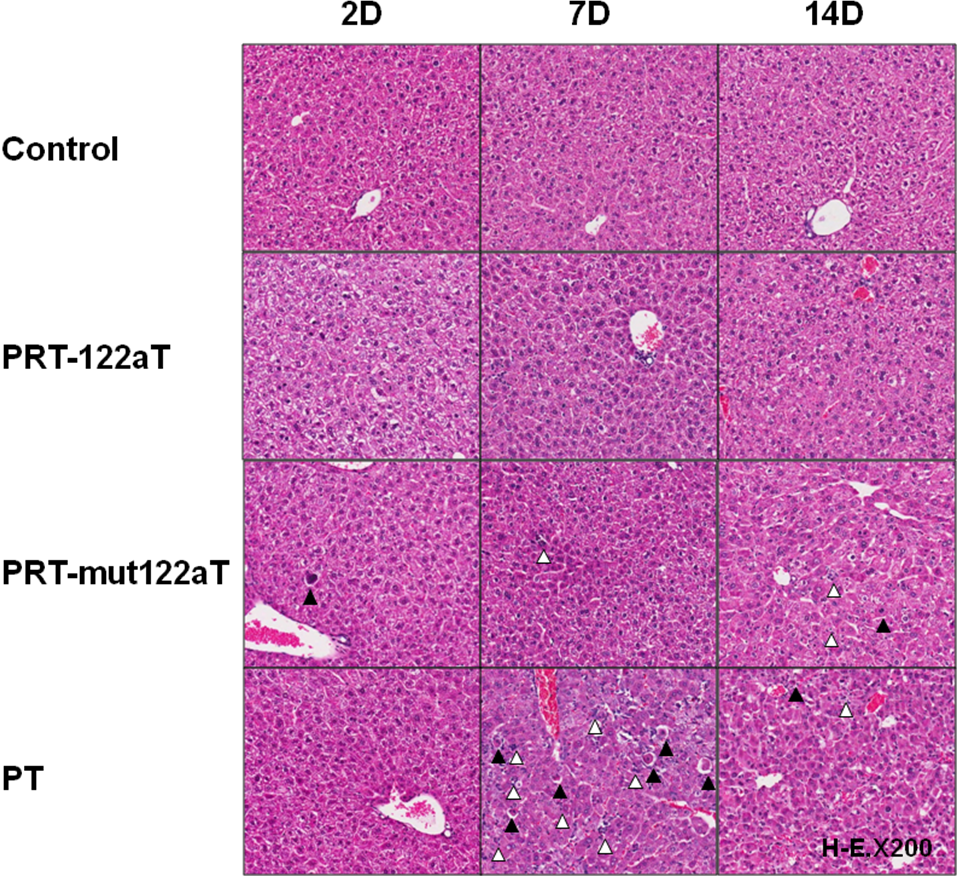

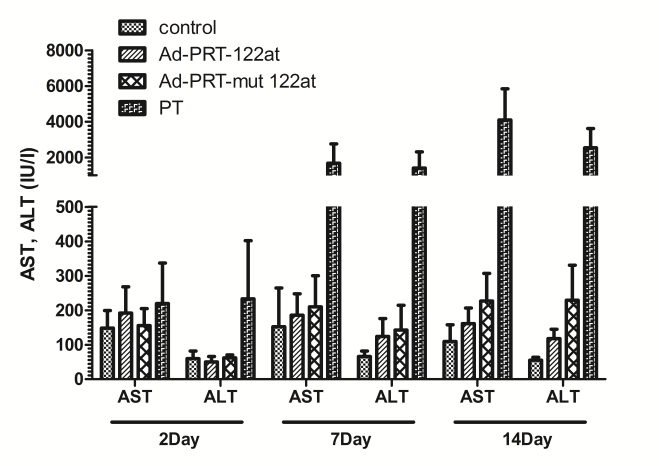
**

**Supplementary Figure 5. Hepatotoxicity of systemically delivered adenoviruses encoding hTERT-targeting ribozyme in normal C57/BL mice.** (A) Representative liver histology of adenoviral-treated mice. Mice were injected with 1 × 10^11^ v.p. of Ad-PRT-122aT, Ad-PRT-mut 122aT, Ad-PT or Ad-PL (control) (*n* = 15 each group) through the tail vein and treated with GCV (50 mg kg^-1^) for 10 days. Five mice from each group were killed on days 2, 7 and 14 after GCV treatment. Liver tissues were processed and paraffin embedded, and the sections were stained with H&E (× 200). The livers infected with Ad-PT showed remarkably frequent apoptosis (filled triangle), spotty necrosis and inflammatory cell aggregates (empty triangle) on day 7, continuing until day 14, which was mainly due to nonspecific HSVtk expression in the normal liver. Ad-PRT-mut 122aT-treated livers showed occasional apoptosis on day 2 and day 7, which was increased on day 14, probably because of leaky expression of HSVtk even though there was no targetable hTERT RNA in the normal mouse liver. In contrast, the livers infected with Ad-PRT-122aT showed no remarkable changes, similar to those treated with control (PL), up until day 14. (B) Liver function of the viral-infected and GCV-treated mice. Liver enzymes, AST and ALT, were measured on days 2, 7 and 14 after GCV inoculation. Results are represented as average ± SD. Significant elevation of liver enzyme levels in the serum was observed in the Ad-PT group from day 2 (ALT) or day 7 (AST) when compared to Ad-PRT-122aT, Ad-PRT-mut 122aT, or the control group. In contrast, the levels of ALT/AST in the Ad-PRT-122aT and Ad-PRT-mut122aT groups were similar to the level of the control group until the 7th day. The ALT/AST levels of Ad-PRT-mut 122aT were higher than Ad-PRT-122aT-treated liver on day 14. These data indicate that insertion of miR-122aT sites to the *trans*-splicing ribozyme reduces the hepatotoxicity caused by improbable nonspecific leaky expression of HSVtk in the liver.

**A**

**
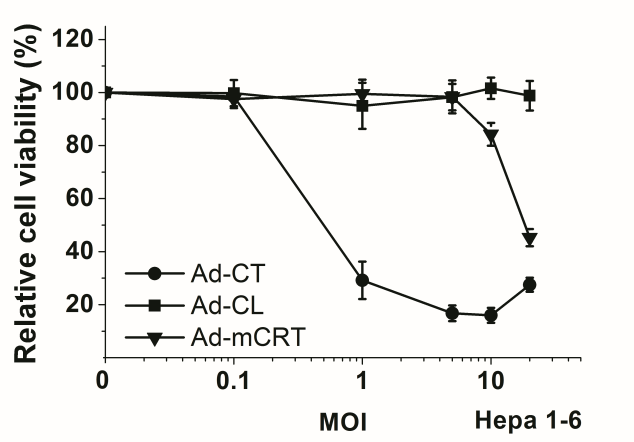
**

**B**

**
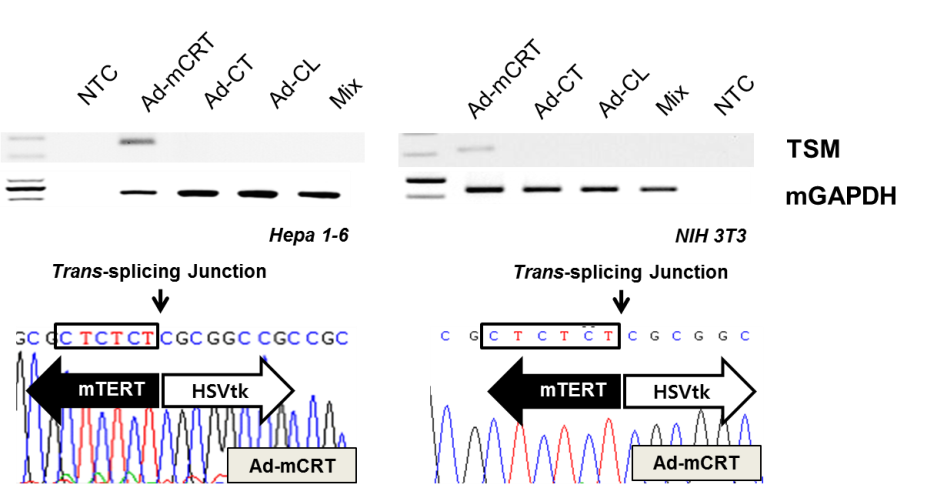
**

**Supplementary Figure 6. Confirmation of mTERT-specific *trans*-splicing ribozyme activity.** (A) Cytotoxicity of the mTERT-targeting *trans-*splicing ribozyme. Hepa 1-6 cells were infected with adenovirus encoding mCRT, CT, or CL at various MOI and inoculated with 100 μM GCV. Cell viability was determined via MTS assay. Results represent the means ± SD of three independent experiments. Ad-mCRT infected cells displayed MOI-dependent cytotoxicity, although with much less efficacy compared with Ad-CT. (B) RNA analysis of the adenoviral-infected cells. Hepa 1-6 and NIH3T3 cells were mock-infected (Mock) or infected with Ad-mCRT, Ad-CT, or Ad-CL at 10 MOI. SKLU-1 (mTERT negative) cells infected with Ad-mCRT were mixed with mock-infected Hepa 1-6 or NIH3T3 cells (mix). TSMs generated in the cells were amplified, yielding a DNA fragment of 177 bp. Mouse GAPDH RNA was amplified as an internal control. Note that cropped gel images are used in this figure and the gels were run under the same experimental conditions. Representative sequences of TSMs generated from Hepa 1-6 or NIH3T3 cells infected with Ad-mCRT were shown.

**A B**

**
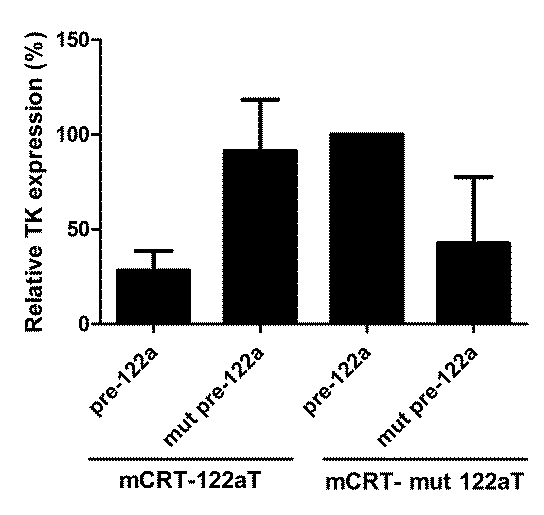
**
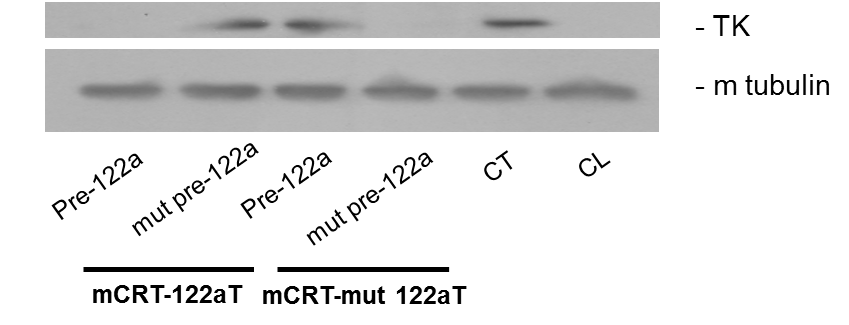


**C**

**
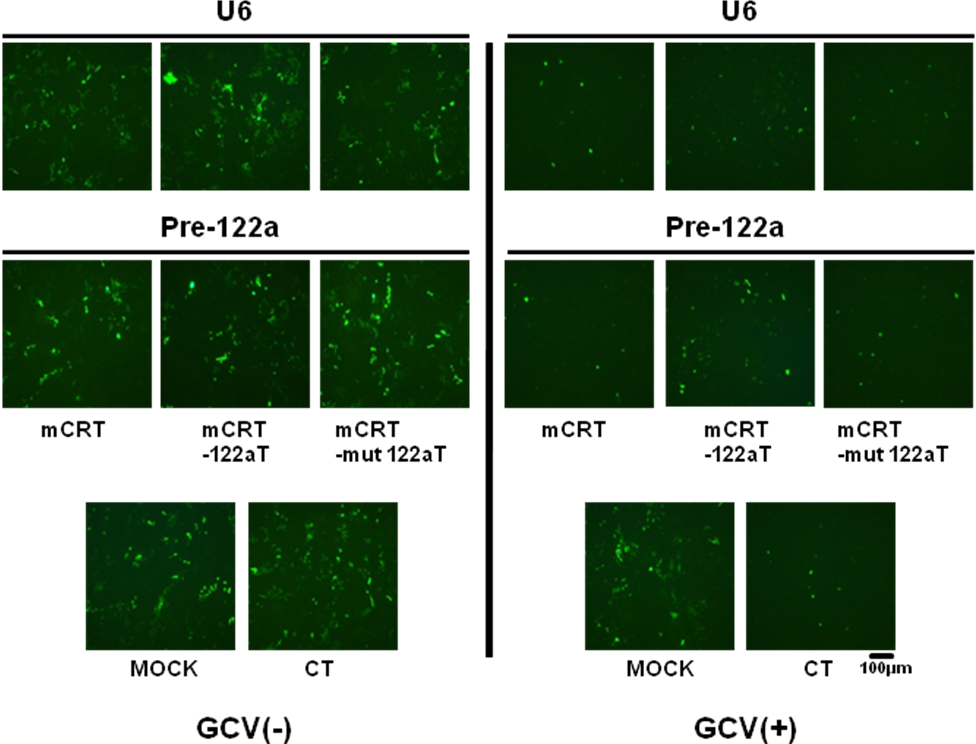
**

**Supplementary Figure 7. Selective regulation of transgene expression of mCRT-122aT by miR-122a.** (A) Relative HSVtk RNA expression levels determined by qRT-PCR. Pre-miR-122a-encoding vector and mCRT-122aT were co-transfected into mTERT(+) Hepa1-6 cells. mCRT-mut 122aT was used as a control. (B) HSVtk protein expression levels by western blotting. Mouse tubulin was loaded for an internal control. Note that cropped gel images are used in this figure and the gels were run under the same experimental conditions. (C) Selective suicide gene induction by the miR-122a-regulated ribozyme. Hepa 1-6 cells were co-transfected with pre-miR-122a, mCRT-122aT, and GFP-encoding vector and treated with 100 μM GCV for 2 days. Cell viability was determined by observing GFP-positive cells after GCV treatment. Cytotoxicity was not affected specifically in cells treated with GCV after co-transfection with mCRT-122aT and pre-miR-122a, indicating that suicide gene activation by the miRNA-regulated ribozyme was controllable through miR-122a expression. Scale bar was shown at bottom right corner.

**A**

**
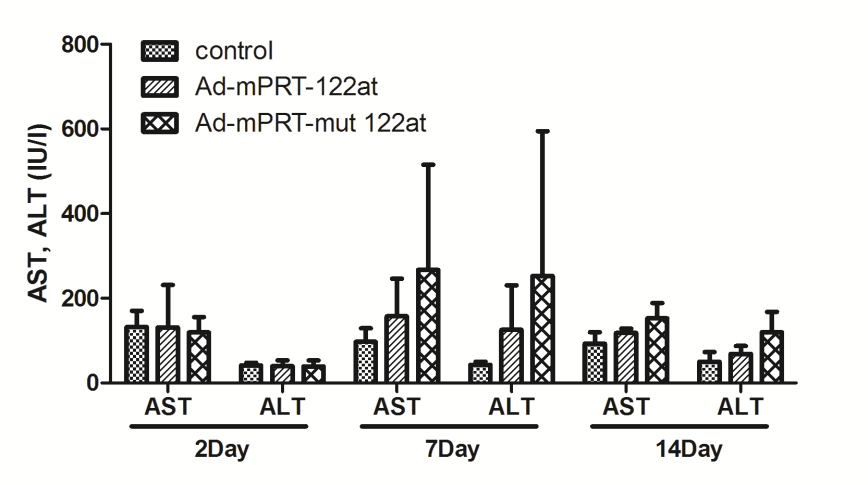
**

**B**

**
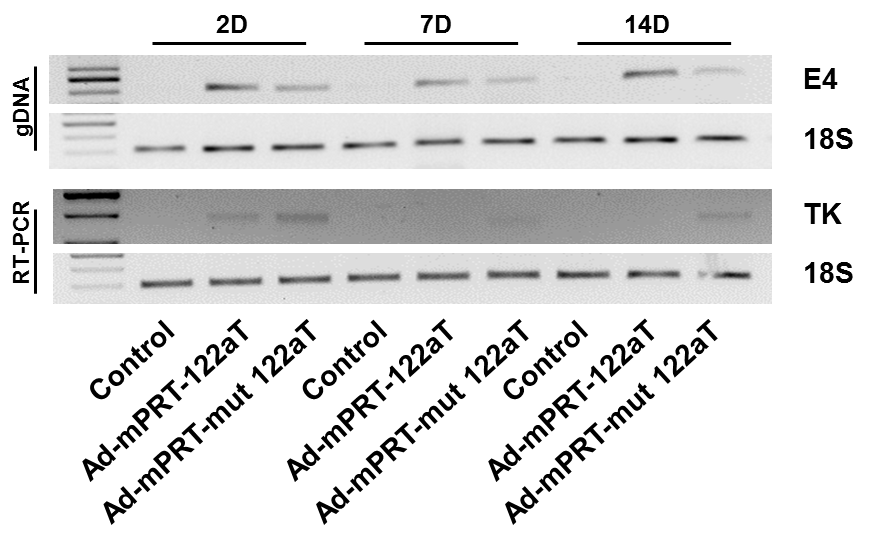

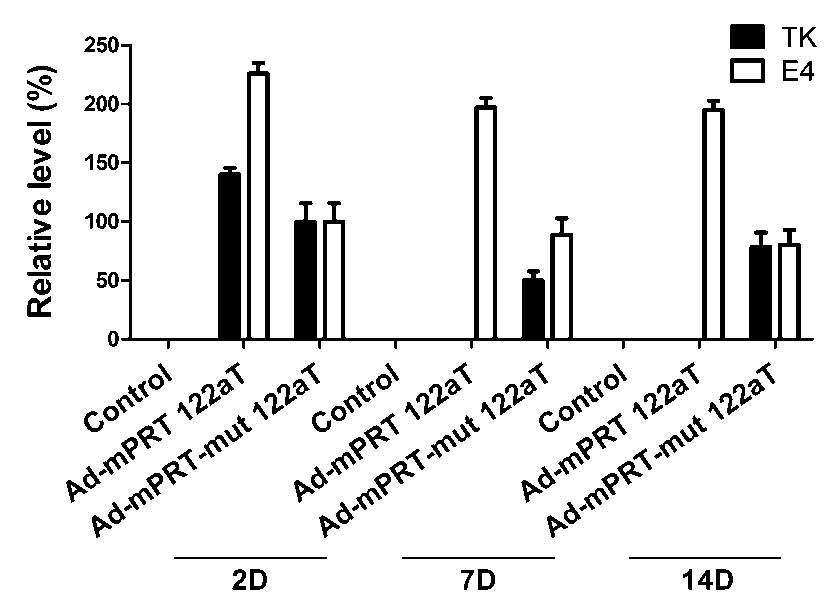
**

**C**

**
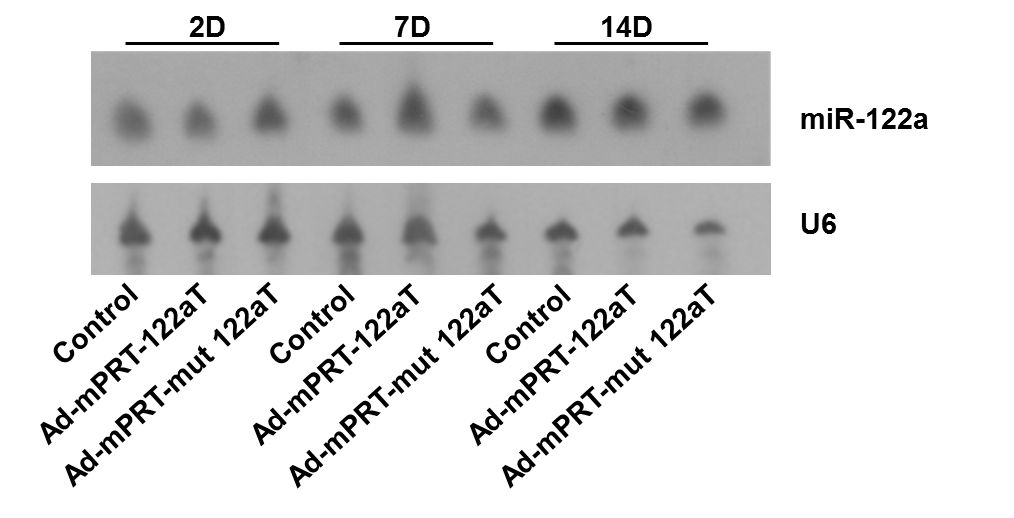
**

**Supplementary Figure 8. Hepatotoxicity and regulated transgene expression of Ad-mPRT-122aT in normal C57/BL mice.** (A) Mice were injected with 1 × 10^11^ v.p. of each adenovirus or PBS (*n* = 15 each group) through the tail vein and treated with GCV (50 mgkg^-1^) for 10 days. Five mice from each group were killed on days 2, 7, and 14 after GCV treatment, and liver enzymes were measured. Results are represented as average ± SD. The liver enzyme levels of Ad-mPRT-122aT-treated liver were lower than Ad-mPRT-mut 122aT-infected liver in the serum on day 7. (B) Ribozyme RNA and viral DNA patterns in the liver of mice injected with adenovirus. Ribozyme production was analyzed through RT-PCR of HSVtk (TK). Infection with adenovirus was confirmed by PCR of E4 genomic DNA. The 18S RNA and 18S genomic DNA were amplified as internal controls. Note that cropped gel images are used in this figure and the gels were run under the same experimental conditions (upper panel). TK RNA level and virus genomic DNA level was measured using real-time PCR in liver from each mice and expressed as a percentage of the level of Ad-mPRT-mut 122aT on day 2. Data are average values ± SD (bottom panel). HSVtk RNA was observed until 14 days post-infection in livers infected with Ad-mPRT-mut 122aT, but disappeared from 7 days post-infection in those treated with Ad-mPRT-122aT, which indicates regulation of ribozyme expression in normal liver by miR-122a. (C) Level of mature miR-122a in the liver tissue of virally-infected mice in (B) was analyzed using northern blot. U6 RNA was used as an internal control. Note that cropped gel images are used in this figure and the gels were run under the same experimental conditions. No difference in the endogenous miR-122a level was observed between Ad-mPRT-122aT and Ad-mPRT-mut 122aT during the infection in the normal livers of C57BL mice.

**A**

**
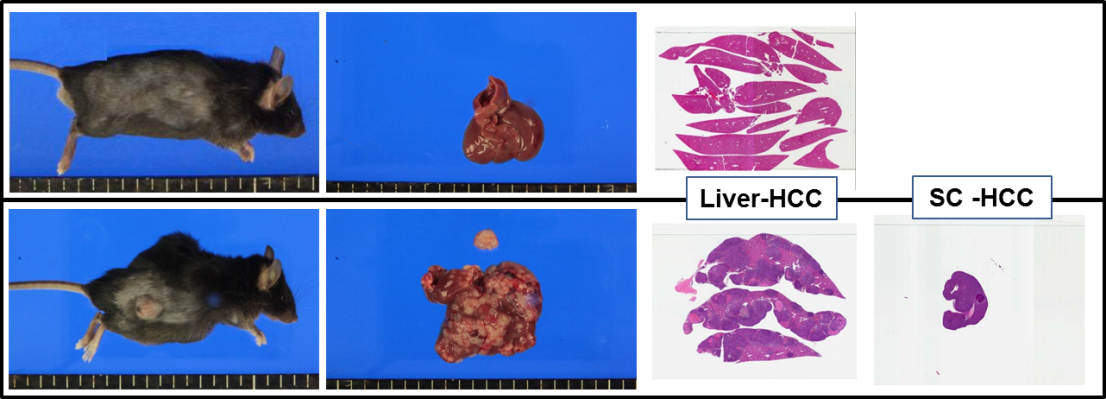
**

**B**

**
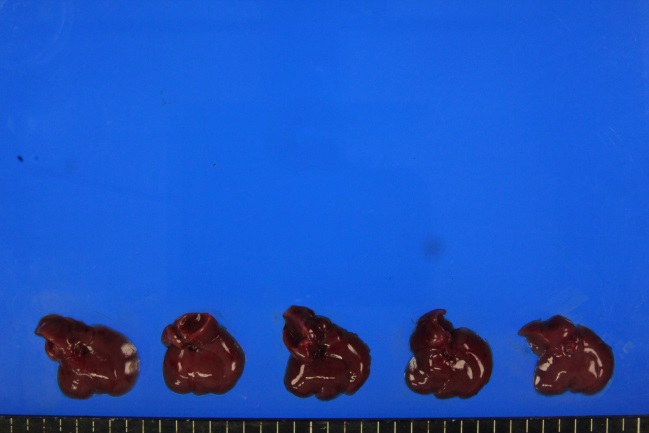
**

**
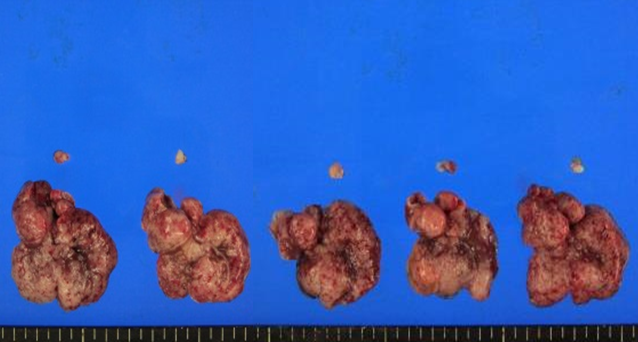
**

**Supplementary Figure 9. Gross and microscopic findings of the challenge assay in Ad-mPRT-122aT/GCV treated syngeneic mouse C57BL model of orthotopic HCC.** (A) Systemic anti-tumor immunity against parental tumor cell challenges at distant sites. Hepa 1-6 cells were inoculated subcutaneously in the flank of mice treated with adenovirus/GCV at different time points after primary splenic subcapsular cell inoculation. The entire livers and subcutaneous tumor mass were photographed under virtual microscope. (B) Photographs of entire livers and subcutaneous tumor mass of each group of mice (*n* = 5). Upper panel: Ad-mPRT-122aT/GCV; bottom panel: control/GCV group.
